# Effect of pre-warming on perioperative hypothermia during holmium laser enucleation of the prostate under spinal anesthesia: a prospective randomized controlled trial

**DOI:** 10.1186/s12871-018-0668-4

**Published:** 2018-12-22

**Authors:** Joo-Hyun Jun, Mi Hwa Chung, Eun Mi Kim, In-Jung Jun, Jung Hwa Kim, Joon-Sang Hyeon, Mi Hyeon Lee, Hye Sun Lee, Eun Mi Choi

**Affiliations:** 1 Department of Anesthesiology and Pain Medicine, Kangnam Sacred Heart Hospital, Hallym University College of Medicine, 1, Shingil-ro, Yeongdeungpo-gu, Seoul, 07441 Republic of Korea; 2 Department of Anesthesiology and Pain Medicine, Hongje Nara Pain Medicine, Seoul, Republic of Korea; 3 Department of Biostatistics, Yonsei University College of Medicine, Seoul, South Korea

**Keywords:** Hypothermia, Pre-warming, Spinal, Temperature

## Abstract

**Background:**

The purpose of this study is to assess whether the application of preoperative forced air warming set to high temperature (> 43 °C) for brief period can increase temperature on admission to the postanesthesia care unit (PACU) and prevent hypothermia or shivering during holmium laser enucleation of the prostate performed under spinal anesthesia.

**Methods:**

Fifty patients were enrolled were assigned randomly to receive passive insulation (control group, *n* = 25) or forced-air skin surface warming for 20 min before spinal anesthesia (pre-warming group, n = 25). The primary outcome was temperature at PACU admission.

**Results:**

The pre-warming group had a significantly higher temperature on admission to the PACU than the control group (35.9 °C [0.1] vs 35.6 °C [0.1], *P* = 0.023; 95% confidence interval of mean difference, 0.1 °C–0.5 °C). The trend of decreasing core temperature intraoperatively was not different between groups (*P* = 0.237), but intraoperative core temperature remained approximately 0.2 °C higher in the pre-warming group (*P* = 0.005). The incidence of hypothermia on admission to the PACU was significantly lower in the pre-warming group (56% vs 88%, *P* = 0.025). Shivering occurred in 14 patients in the control group, and 4 patients in the pre-warming group (*P* = 0.007).

**Conclusion:**

Brief pre-warming at 45 °C increased perioperative temperature and decreased the incidence of hypothermia and shivering. However, it was not sufficient to modify the decline of intraoperative core temperature or completely prevent hypothermia and shivering. Continuing pre-warming to immediately before induction of spinal anesthesia or combining pre-warming with intraoperative active warming may be necessary to produce clearer thermal benefits in this surgical population.

**Trial registration:**

This trial was registered with Clinicaltrials.gov, NCT03184506, 5th June 2017.

## Background

General anesthesia greatly impair central thermoregulation, reducing the thresholds for vasoconstriction and shivering [1, 2]. Consequently, most anesthetized patients who did not receive active warming become hypothermic [3]. Neuraxial anesthesia impairs central thermoregulatory control less than does general anesthesia [4]. However, unlike general anesthesia, neuraxial anesthesia blocks peripheral sympathetic and motor nerve, which inhibits thermoregulatory vasoconstriction and shivering in blocked area [5]. Thus, perioperative hypothermia during neuraxial anesthesia is common and severe as during general anesthesia [6]. Furthermore, as neuraxial anesthesia impair behavioral thermoregulatory responses (i.e., patient sensation of cold) and routine core temperature monitoring remains rare during regional anesthesia, substantial hypothermia commonly goes undetected [7].

Holmium laser enucleation of prostate (HoLEP), which utilizes laser energy and normal saline irrigation, is a minimally invasive alternative to transurethral resection of the prostate (TURP) and being performed with increasing frequency [8]. Patients undergoing HoLEP have a high risk of hypothermia because large volumes of cold irrigation fluid reduce core body temperature by 1 °C to 2 °C [9]. In addition, most patients undergoing HoLEP are elderly, which is the most significant contributor of hypothermia in both neuraxial and general anesthesia [10, 11]. Use of isothermic irrigation fluid has been shown to be efficacious in reducing heat [12, 13]. However, it is difficult to warm a large volume of irrigation fluid without continuous warming device, and burning of tissue can be caused by instillation of over-warming irrigation fluid [9].

Heat redistribution from the core to the periphery by vasodilation is the most important cause of perioperative hypothermia during the first hour after induction of general or neuraxial anesthesia [5, 14, 15]. The amount of redistribution depends on the core temperature gradient between the peripheral and core compartments. Actively warming the skin surface’s before surgery (i.e., pre-warming) can reduce this gradient by increasing the heat content of the peripheral thermal compartment [16]. Current clinical guidelines for prevention of inadvertent perioperative hypothermia recommend pre-warming for both neuraxial and general anesthesia [17]. It was also reported that only 20 min of pre-warming at a high temperature (44 °C) generally prevented perioperative hypothermia in general populations undergoing general anesthesia [16]. However, the benefits of a short period of pre-warming at a high temperature have not been examined in elderly patients at high risk of hypothermia undergoing neuraxial anesthesia.

The purpose of this study is to assess whether the application of preoperative forced air warming set to high temperature (> 43 °C) for brief period can increase temperature on admission to the postanesthesia care unit (PACU) and prevent hypothermia or shivering during HoLEP performed under spinal anesthesia.

## Methods

This prospective, single-blind, randomized, controlled study was approved by the Institutional Review Board at Hallym University Kangnam Sacred Heart Hospital (reference number 2017–05-002). All participants provided written informed consent. The trial was registered prior to patient enrollment at ClinicalTrials.gov (NCT03184506). The study was conducted and reported in accordance with the Consolidating Standards of Reporting Trials (CONSORT) 2010 statement [18]. Patients were recruited from June 2017 to March 2018.

Patients aged 50 to 80 years, with an American Society of Anesthesiologists’ physical status class of I to III and undergoing HoLEP under spinal anesthesia were included in the study. The exclusion criteria were a preoperative core temperature > 37.2 °C; severe endocrine, cardiovascular, or respiratory disease; or contraindication to spinal anesthesia (due to bleeding diathesis, neurologic dysfunction or recent local infection).

Patients were randomly assigned in a 1:1 ratio to the pre-warming group or control group. A random allocation sequence was created by one of the investigators (EMC) using a computer-generated randomization schedule (http://www.randomizer.org). On arrival in the preoperative holding room, another investigator (JHJ), who was not involved in data collection, opened an opaque, sealed envelope that contained the patient’s group assignment.

### Study protocol

No premedication was given to any patient. Preoperative, intraoperative, and postoperative ambient temperatures were kept between 22 °C and 24 °C.

In the preoperative holding area, participants in the control group received usual care; they were covered with only two layers of a warmed cotton blanket, which were stored in a warming cabinet (KRS-205; Karis, Gyeonggi-do, Korea) set to 41 °C until immediately before use, and received no active warming. Participants in the pre-warming group received 20 min of active warming with a forced-air blanket (COVIDIEN™ WarmTouch™ Full Body/Multi Access Blanket, Covidien PLC, Mansfield, MA, USA), which was placed over the entire body and then covered with a cotton blanket. During the warming period, the forced-air warmer (COVIDIEN™ WarmTouch™ WT6000 Warming Unit, Covidien PLC) was set to “high” (45 °C). The participants were queried about their thermal comfort every 5 min during warming, and the temperature was reduced to 41 °C if they indicated feeling too warm. At the end of active warming, the forced-air warming blanket was removed and replaced with two layers of a warmed cotton blanket.

Upon entry in the OR, all participants received an intravenous (IV) bolus of 8 mL/kg Ringer’s lactate as a preloading fluid, which was followed by a continuous infusion of IV Ringer’s lactate at 2 mL/kg/hr. All IV fluids used during surgery were stored at room temperature, in accordance with our routine practice. Spinal anesthesia was induced in the lateral decubitus position by an investigator blinded to the group allocation (JHK). After skin infiltration with lidocaine, dural puncture was performed using a 25-gauge Whitacre spinal needle at L3–4 or L4–5. After return of free-flowing, clear cerebrospinal fluid, 0.5% hyperbaric bupivacaine 13 mg was injected into the subarachnoid space; in patients with height < 160 cm or > 180 cm, the dose was reduced or increased. The extent of sensory blockade was tested by the pinprick method, and anesthesia was considered adequate if the sensory block was at the T8 dermatomal level or higher. Hypotension (systolic blood pressure lower than 80% of the baseline value or 90 mmHg) was treated with phenylephrine (30–50 μg) or ephedrine (4 mg) and repeated as necessary. After the induction of anesthesia, patients were placed in the lithotomy position. All of the HoLEP surgeries were performed by the same urologic team. Normal saline irrigation was used for visualization during the entire surgical procedure (standard in HoLEP). Total volume of irrigation fluid is documented. All patients were covered with two layers of a warmed cotton blanket from the neck to the umbilicus to protect against heat loss; no active heating was planned intraoperatively. However, the forced-air warmer system was applied to the upper body if patients requested warming.

In the PACU, the forced-air warmer system was applied over the whole body if the core temperature was less than 36 °C or if the patient acknowledged feeling cold or was shivering.

### Outcome measurements

One investigator (JHK) blinded to the group allocation evaluated the all perioperative outcomes except core temperature on arrival in the preoperative holding area.

Core temperatures were measured using an infrared tympanic thermometer (ThermoScan IRT 1020; Braun, Germany). The device accuracy mean error was found to be ±0.2 °C when patients temperature 35.8 to < 37 °C and ± 0.3 °C when patients temperature < 35.8 °C. The same ear was used for all measurements, and the highest of three consecutive temperatures was recorded. The core temperature was recorded at these time points: on arrival in the preoperative holding area (preoperative), on arrival in the OR, immediately after the induction of spinal anesthesia, every 30 min intraoperatively after the induction of anesthesia, and on admission to the PACU. Hypothermia was defined as a core temperature below 36.0 °C, in accordance with current guidelines [17]. The number of participants with hypothermia at PACU admission was recorded.

Shivering was rated according to a 4-point scale: 0, none; 1, only in the neck and core; 2, including the upper extremities; and 3, involving the entire body [19]. Patients with shivering scores ≥3 after induction of spinal anesthesia received IV meperidine 25 mg. Thermal comfort was assessed using a continuous numeric rating scale: 0, extremely cold; 50, thermally neutral; and 100, extremely hot. Shivering and thermal comfort were evaluated at these time points: on arrival in the OR, every 30 min after induction of spinal anesthesia, on arrival in the PACU, and every 30 min after PACU arrival for a total of two times. For all patients, we recorded age, height, and weight; length of anesthesia and surgery; and volume of IV and irrigation fluids received intraoperatively.

The primary outcome was core temperature on arrival in the PACU. Secondary outcomes included changes in temperature from arrival in the OR until the end of surgery, incidence of hypothermia on admission to the PACU, perioperative shivering (incidence, severity, and meperidine use), and perioperative thermal comfort scores.

### Statistical analyses

We used SPSS 24.0 (IBM, Armonk, NY) or SAS version 9.3 (SAS Institute, Cary, NC) for all statistical analyses. The normality of continuous data was assessed with the Shapiro–Wilk test. Balance in baseline variables across the two groups was assessed by their effect size. The effect size was assessed by calculating the standardized difference, which was the difference in means or proportions divided by the pooled standard deviation. We considered a standardized difference > 0.20 as evidence of sufficient imbalance to require adjustment in subsequent analyses.

The primary outcome (temperature on arrival in the PACU) was assessed using Student’s t test for independent groups and reported with a 95% confidence interval (CI) and *P* value.

Core temperature changes intraoperatively were assessed with a linear mixed model using the SAS MIXED procedure (version 9.3; SAS Institute, Cary, NC) and restricted maximum likelihood estimation. The fixed effects were time of assessment, treatment (control and pre-warming), and treatment-by-time interaction. We evaluated the interaction between treatment group and time adjusted for preoperative core temperature because a treatment-by-time interaction reflects differing temperature changes over time based on treatment. The overall mean difference in core temperature between groups was further evaluated using a mixed effects model with repeated measures. We also conducted post hoc analyses to determine the times when treatment effects differed between groups. In these analyses, the least square means of both groups were estimated at each time using the MIXED procedure and compared using two-sample t tests with Bonferroni correction. Corresponding CIs were appropriately adjusted for multiple comparisons using the Bonferroni correction.

Non-normally distributed continuous outcomes, such as shivering and thermal comfort scores, were analyzed using Mann–Whitney tests. Categorical outcomes (incidence of hypothermia, shivering, meperidine use, and need for active warming in the PACU) were analyzed using the Pearson chi-square or Fisher’s exact tests, as appropriate. In all analyses, *P* < 0.05 was considered statistically significant.

The sample size was calculated based on the primary outcome (temperature on arrival in the PACU) using G*Power (version 3.0.10, Franz Faul, Universitat Kiel, Germany). A 0.5 °C difference in postoperative core temperature was considered clinically important because 0.5 °C is the smallest difference associated with hypothermia-related complications [20]. Twenty-two patients in each group were necessary to detect this difference with 90% power and a significance level of 5%, using two-sided tests. Therefore, 25 patients per group were enrolled to compensate for a possible 10% dropout rate.

## Results

**Fig. 1:**
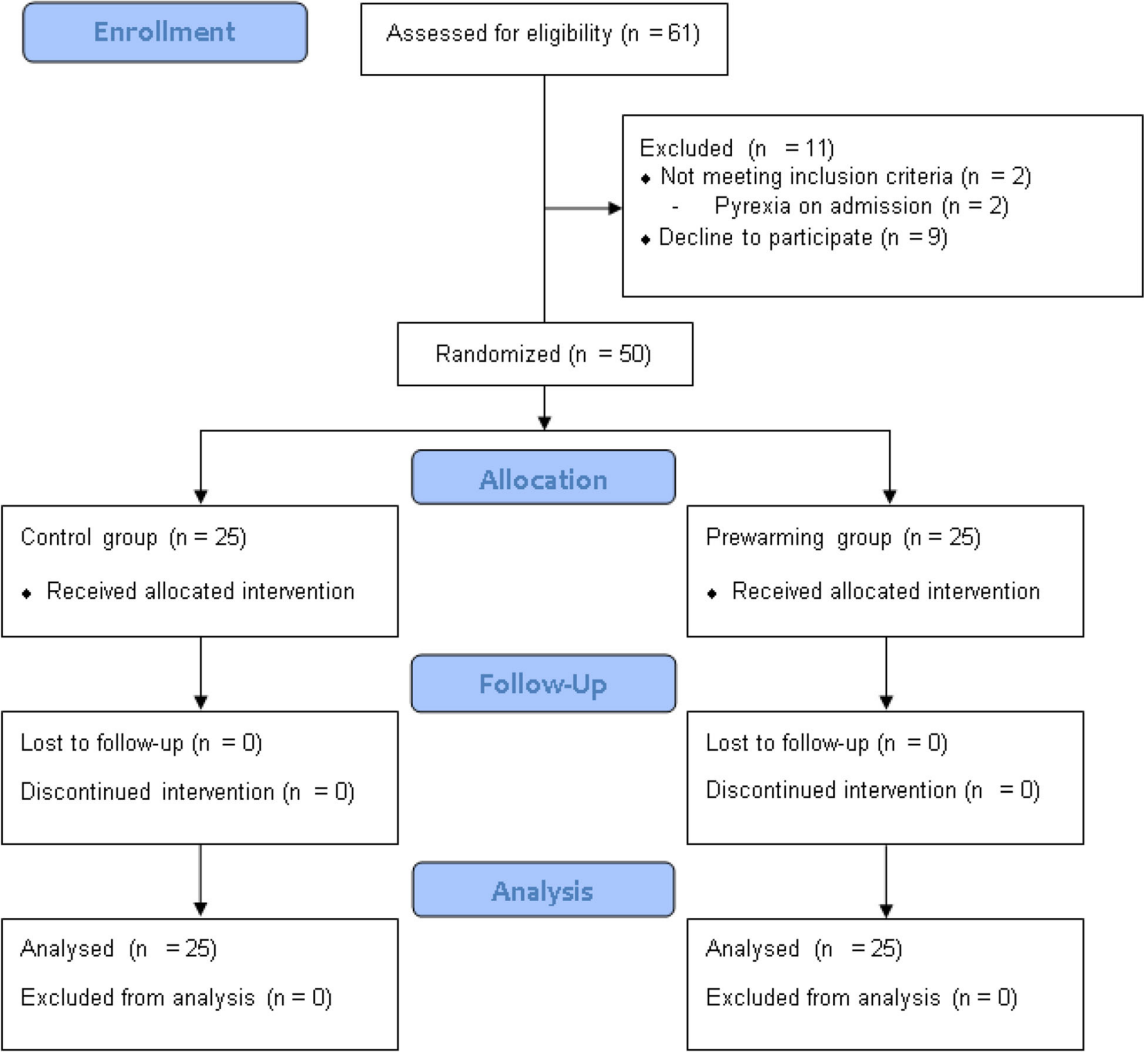
CONSORT flow diagram

A total of 61 patients were screened; 2 failed to meet the inclusion criteria, and 9 declined to participate. The 50 eligible patients were randomized (25 patients in each group) and included in the final analysis (Fig. 1). The two groups were poorly balanced for preoperative core temperature, with a standardized difference of 0.63 (Table 1). We therefore adjusted for preoperative temperature when comparing intraoperative and postoperative core temperatures between groups. Other demographic and perioperative data were sufficiently balanced across the two groups (Table 1). Anesthesia duration was between 60 and 210 min, and mean surgery duration was similar in both groups. All patients tolerated the preoperative warming well; no patient requested lowering the temperature to 41 °C.

**Table 1:** Patients’ Baseline, Surgical, and Anesthetic Data

	Control	Pre-warming	Standardized difference^c^
	(*n* = 25)	(*n* = 25)	
Age (y)	65 ± 7	66 ± 7	0.106
Body mass index (kg/m^2^)	25.2 ± 2.2	25.3 ± 2.5	0.042
Sensory block level	T8 (T6–T8)	T6 (T6–T8)	0.146
Intrathecal bupivacaine (mg)	13 (13–13.5)	13 (13–13.5)	0.044
Duration of anesthesia (min)	95 (80–133)	100 (75–118)	0.112
Duration of operation (min)	60 (53–95)	65 (48–85)	0.171
Spinal time (min)^a^	10 (10–10)	10 (8–10)	0.022
Clean up time (min)^b^	19 (15–21.5)	20 (16–24)	0.151
Resected prostate (g)	40 (30–50)	40 (20–60)	0.007
Total irrigation fluid (L)	21 (18–33)	24 (1.5–3)	0.192
Total intravenous fluid (mL)	730 ± 157	742 ± 147	0.079
Preoperative core temperature (°C)	36.8 ± 0.1	36.7 ± 0.2	0.632

Values are mean ± standard deviation (SD) or median (interquartile range)

^a^Spinal time: The time from arrival in the operation room to immediately after the induction of spinal anesthesia

^b^Clean up time: The time from immediately after the induction of spinal anesthesia to the start of surgery

^c^Difference in means or proportions divided by pooled SD

Variables with a standardized difference > 0.20 were considered unbalanced and adjusted for in subsequent analyses

### Primary outcome

**Fig. 2:**
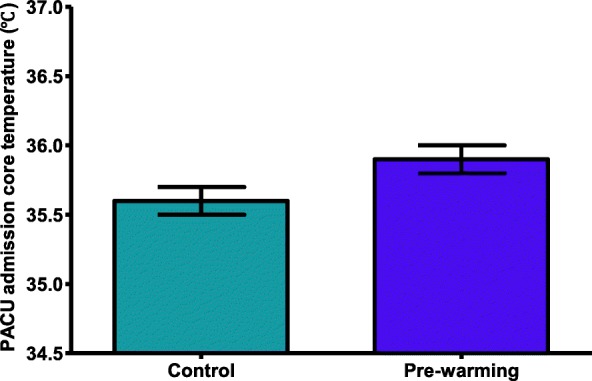
Core temperature upon entering the postanesthesia care unit. Boxes represent the estimated mean values in minutes, and whiskers represent ± standard error of the mean. *P* = 0.027 between control and pre-warming groups

Temperature on arrival in the PACU was significantly higher in the pre-warming group than in the control group. The estimated mean (standard error) temperature was 35.9 (0.1)°C in the pre-warming group and 35.6 (0.1)°C in the control group (difference, 0.3 [95% CI, 0.1–0.5]; *P* = 0.027) (Fig. 2).

### Secondary outcomes

**Fig. 3:**
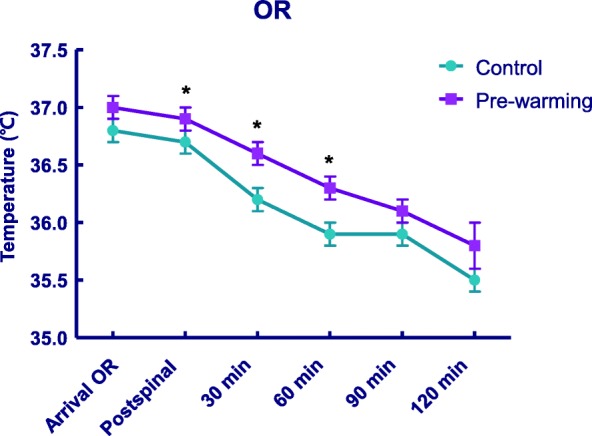
Serial changes in core temperature in the operating room (OR). Values are mean and standard error of the mean. Core temperature changes were not significantly different between the two groups (*P* = 0.237). * *P* < 0.05; mean core temperatures differed significantly between control and pre-warming groups from immediately after induction of spinal anesthesia to 60 min after induction

Intraoperative core temperature changes are shown in Fig. 3. No significant treatment-by-time interaction was observed, suggesting that the trend of decreasing core temperature intraoperatively did not differ between the control and pre-warming groups (*P* = 0.237) (Fig. 3). However, the overall mean difference in core temperature during surgery between the two groups was 0.2 °C (95% CI, 0.1–0.4, *P* = 0.005), suggesting intraoperative core temperature remained approximately 0.2 °C higher in the pre-warming group (Table 2). In post hoc analyses, the pre-warming group exhibited a higher mean core temperature from immediately after the induction of spinal anesthesia until 60 min after induction (Table 2) (Fig. 3).

**Table 2:** Core Temperature (in °C) at Each Assessment Time in the Operating Room

Time	Control	n	Pre-warming	n	Difference (CI)^b^	*P* value^c^
On arrival in the operation room	36.8 (0.1)	25	37.0 (0.1)	25	0.2 (−0.1–0.4)	0.119
Immediately after spinal induction	36.7 (0.1)	25	36.9 (0.1)	25	0.2 (0.1–0.4)	0.029
30 min after spinal induction	36.2 (0.1)	25	36.6 (0.1)	25	0.3 (0.1–0.6)	0.006
60 min after spinal induction	35.9 (0.1)	23	36.3 (0.1)	23	0.4 (0.1–0.7)	0.014
90 min after spinal induction	35.9 (0.1)	9	36.1 (0.1)	13	0.2 (−0.2–0.5)	0.706
120 min after spinal induction	35.5 (0.1)	7	35.8 (0.2)	4	0.2 (−0.3–0.8)	> 0.999
Overall effect^a^					0.2 (0.1–0.4)	0.005

^a^Overall mean difference in core temperatures between control and pre-warming groups intraoperatively

^b^Confidence intervals (CIs) were adjusted for multiple comparisons using the Bonferroni correction; 95% CI was reported for the overall effect, and 99.2% CI was reported at each assessment time (i.e., 0.05/6)

^c^*P* values were corrected using the Bonferroni method for multiple comparisons

On arrival in the PACU, more patients in the control group (88%) were hypothermic than in the pre-warming group (56%; *P* = 0.025) (Table 3). The overall incidences of perioperative shivering and meperidine use were significantly lower in the pre-warming group than in the control group (*P* = 0.007 and 0.016, respectively). The lowest perioperative thermal comfort scores and the need for active warming in the PACU did not differ between groups (Table 3).

**Table 3:** Perioperative Outcomes

Outcome	Control	Pre-warming	*P* value
	(*n* = 25)	(*n* = 25)	
Hypothermia at PACU arrival	22 (88)	14 (56)	0.025
Perioperative shivering	14 (56)	4 (16)	0.007
Perioperative meperidine use	13 (52)	4 (16)	0.016
Shivering score (from 0 to 3)			
Intraoperative	0 (0–0)	0 (0–0)	0.317
PACU	0 (0–1)	0 (0–0)	0.035
Thermal comfort score (from 0 to 100)			
Intraoperative	40 (38–50)	40 (30–50)	0.407
PACU	35 (30–40)	40 (30–50)	0.340
Active warming required in the PACU	23 (92)	21 (88)	0.667

Values are number of patients (proportion) or median (interquartile range)

Shivering scores were based on a 4-point numerical scale: 0, none; 1, localized to the neck and core; 2, including the upper extremities; and 3, involving the entire body. Thermal comfort scores were based on a continuous verbal numerical scale: 0, extremely cold; 50, thermally neutral; and 100, extremely hot

*PACU* postanesthesia care unit

## Discussion

Twenty minutes of pre-warming at 45 °C was associated with a significantly higher core temperature—by approximately 0.3 °C—on arrival in the PACU after HoLEP under spinal anesthesia. Pre-warming also produced an approximately 0.2 °C higher temperature intraoperatively than control treatment. However, the trends of decreasing temperature after induction of spinal anesthesia were not modified by pre-warming. Similarly, although pre-warming decreased the rate of perioperative hypothermia and shivering, approximately half of patients receiving this treatment still became hypothermic, and shivering was not completely prevented.

Thermal benefit of brief periods of pre-warming (only 10 to 20 min) at a high temperature (44 °C) has proven in patients undergoing general anesthesia through Horn el al.’s study [16]. Recently, Jo et al. [21] have evaluated the effect of 20 min period of pre-warming in patients undergoing TURP under spinal anesthesia, and cannot decrease the rate of hypothermia or shivering, although it reduced the severity of hypothermia. They explained that their poor outcome was due to age-related decreases in thermoregulatory functions. However, the study applied pre-warming in moderate temperature settings (38 °C). As the extent of heat redistribution depends on the core-to-periphery temperature gradient [22], we assumed that the poor thermal benefit of this study also could be attributed to an insufficient increase of peripheral heat contents due to pre-warming at moderate temperature setting. In the current study, we performed pre-warming at a high temperature (45 °C) to increase the heat content of the peripheral compartment as much as possible in a short period of time. As a result, despite the surgical setting similar to that of Jo et al.’s study (advanced age and the need for large volume of bladder irrigation), current study show thermal benefit including higher temperature on admission to PACU and in the OR, as well as a lower incidence of perioperative hypothermia and shivering.

Our pre-warming intervention decreased the incidence of hypothermia and shivering but did not improve thermal comfort. This result may be caused by tolerance to hypothemia due to an increase in apparent lower body skin temperature with neuraxial anesthesia [23, 24]. In addition, because patients with shivering scores ≥3 immediately managed with IV meperidine 25 mg, shivering also may not have caused the patient’s thermal discomfort to less than expected. Although thermal comfort score was similar to the treatment group, without pre-warming, almost all (88%) of patients undergoing HoLEP under spinal anesthesia became hypothermic on arrival in the PACU. Even mild perioperative hypothermia (34 °C–36 °C) can adversely affect surgical outcomes by increasing the risk of postoperative shivering, cardiac morbidity coagulopathy and transfusion requirement, and surgical site infections [25–29]. Furthermore, shivering can disturb the surgical field, which may result in prolongation of the operation time or severe complications. Therefore, even though pre-warming did not improve thermal comfort in patients undergoing HoLEP, it can be considered that there is a thermal benefit of pre-warming in these surgical populations.

Nevertheless, the overall thermal benefit of pre-warming in our study was modest. Temperature decline trends were not modified, 56% of pre-warming patients became hypothermic, and 16% of pre-warming patients exhibited shivering. The study was designed to determine the effectiveness of a short time pre-warming at high temperature itself on perioperative core temperature. Pre-warming was applied in the preoperative holding area before patients entered the OR. After pre-warming, the patients were treated with only passive insulation (cotton blanket); active warming was performed only at the patients’ request. A time delay between the end of pre-warming and induction of spinal anesthesia (approximately 10–15 min) may have attenuated thermal benefits of pre-warming.

Meanwhile, previous studies of short pre-warming periods (15–30 min) combined with intraoperative active cutaneous warming have reported clear thermal benefits (modification of temperature decline trends and prevention of almost all hypothermia), unlike the results of our study [30–32]. Although heat redistribution is the most important cause of perioperative hypothermia, surgical factors increasing systemic heat loss decrease the relative contribution of redistribution to perioperative hypothermia [22]. Thus, pre-warming alone may be insufficient to prevent hypothermia because large volumes of cold irrigation fluid increase systemic heat loss during HoLEP surgery [33]. As passive insulation reduces only cutaneous heat loss to compensate for anesthesia-induced reduction in metabolic heat production [34], it may be necessary to continue active warming into the intraoperative period to achieve clear thermal benefits in this surgical population. Further studies are necessary to investigate this possibility.

There are limitations to the current study. One is that we did not measure the mean body temperature and therefore could not calculate the actual heat content. However, core temperature is the single temperature that best characterizes a patient’s thermal status, and it is the main outcome measured in clinical practice. Another potential limitation involves the use of infrared tympanic thermometers. The reliability of these thermometers for precisely measuring core temperature has been questioned [35, 36]. However, tympanic contact thermistors and thermocouple ear thermometry are not widely used in clinical practice. Additionally, invasive core temperature monitors, such as esophageal probes, are difficult to use in patients undergoing spinal anesthesia. However, infrared systems can precisely measure surface temperatures, and the measurement site rather than the device determines the precision and accuracy [34]. Furthermore, we recorded the highest of three consecutive measurements from the same ear to reduce measurement error, and it is likely that potential bias introduced by using infrared tympanic thermometers was equally distributed between the two groups. Finally, the study population consisted of only a small number of highly selected patients undergoing HoLEP performed under spinal anesthesia. In HoLEP, cutting and coagulation occur at the same time and thus smaller blood vessels are sealed off instantaneously, decreasing blood loss and intravascular absorption of irrigation fluids [37]. As intravascular absorption of irrigation fluid is less with HoLEP as compared with standard TURP, efficacy of pre-warming in reducing heat loss during HoLEP could differ TURP and HoLEP. However, because anesthetic-induced impairment of thermoregulation is much more important contributor to perioperative hypothermia than cold exposure [22], we assume that a similar thermal benefit of pre-warming could be also found in TURP performed under spinal anesthesia.

## Conclusions

In conclusion, 20 min of pre-warming at 45 °C before spinal anesthesia for HoLEP increased core temperature on admission to the PACU and during the operation and decreased the rates of perioperative hypothermia and shivering. However, pre-warming was unable to modify the decline of core temperatures intraoperatively or completely prevent hypothermia and shivering. In procedures with anticipated excessive heat loss, it may be necessary to continue pre-warming until immediately before the induction of spinal anesthesia or to combine pre-warming with intraoperative active warming to most effectively prevent hypothermia.

## Abbreviations

CI: confidence interval
HoLEP: holmium laser enucleation of the prostate
IV: intravenous
OR: operating room
PACU: postanesthesia care unit
TURP: transurethral resection of the prostate
